# Retrospective evaluation of interval breast cancer screening mammograms by radiologists and AI

**DOI:** 10.1007/s00330-025-11833-5

**Published:** 2025-08-04

**Authors:** Jonas Subelack, Rudolf Morant, Marcel Blum, Axel Gräwingholt, Justus Vogel, Alexander Geissler, David Ehlig

**Affiliations:** 1 Chair of Health Economics, Policy and Management, School of Medicine, University of St.Gallen, St.Gallen, Switzerland; 2 Cancer League of Eastern Switzerland, St.Gallen, Switzerland; 3 Mammographiescreening-Zentrum Paderborn, Paderborn, Germany

**Keywords:** Breast, Breast neoplasms, Mammography, Artificial intelligence, Interval breast cancer

## Abstract

**Objectives:**

To determine whether an AI system can identify breast cancer risk in interval breast cancer (IBC) screening mammograms.

**Materials and methods:**

IBC screening mammograms from a Swiss screening program were retrospectively analyzed by radiologists/an AI system. Radiologists determined whether the IBC mammogram showed human visible signs of breast cancer (potentially missed IBCs) or not (IBCs without retrospective abnormalities). The AI system provided a case score and a prognostic risk category per mammogram.

**Results:**

119 IBC cases (mean age 57.3 (5.4)) were available with complete retrospective evaluations by radiologists/the AI system. 82 (68.9%) were classified as IBCs without retrospective abnormalities and 37 (31.1%) as potentially missed IBCs. 46.2% of all IBCs received a case score ≥ 25, 25.2% ≥ 50, and 13.4% ≥ 75. Of the 25.2% of the IBCs ≥ 50 (vs. 13.4% of a no breast cancer population), 45.2% had not been discussed during a consensus conference, reflecting 11.4% of all IBC cases. The potentially missed IBCs received significantly higher case scores and risk classifications than IBCs without retrospective abnormalities (case score mean: 54.1 vs. 23.1; high risk: 48.7% vs. 14.7%; *p* < 0.05). 13.4% of the IBCs without retrospective abnormalities received a case score ≥ 50, of which 62.5% had not been discussed during a consensus conference.

**Conclusion:**

An AI system can identify IBC screening mammograms with a higher risk for breast cancer, particularly in potentially missed IBCs but also in some IBCs without retrospective abnormalities where radiologists did not see anything, indicating its ability to improve mammography screening quality.

**Key Points:**

***Question*** *AI presents a promising opportunity to enhance breast cancer screening in general, but evidence is missing regarding its ability to reduce interval breast cancers.*

***Findings*** *The AI system detected a high risk of breast cancer in most interval breast cancer screening mammograms where radiologists retrospectively detected abnormalities.*

***Clinical relevance*** *Utilization of an AI system in mammography screening programs can identify breast cancer risk in many interval breast cancer screening mammograms and thus potentially reduce the number of interval breast cancers.*

**Graphical Abstract:**

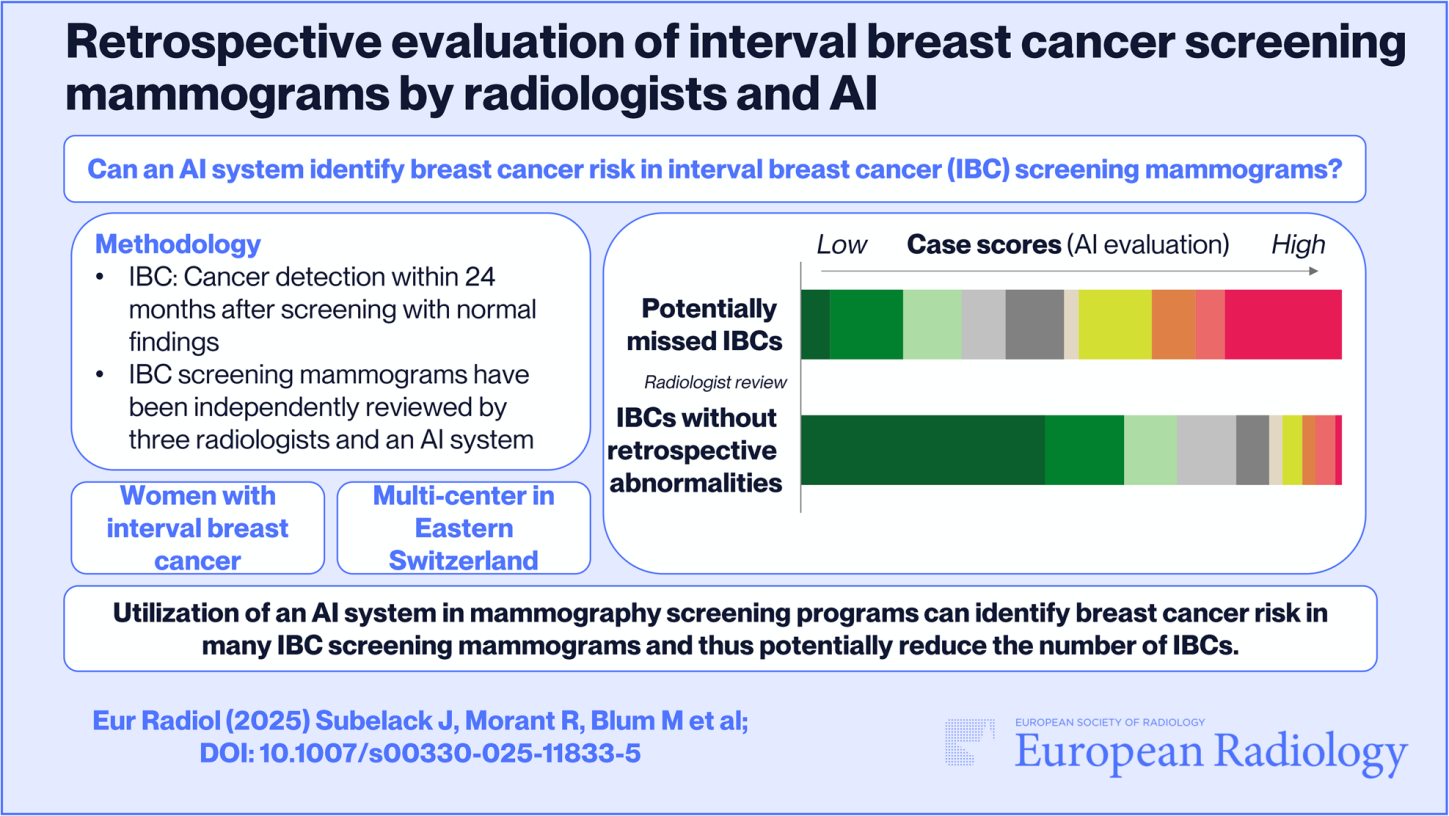

## Introduction

Research demonstrated that participation in a mammography screening program (MSP) leads to a reduction in breast cancer (BC) related mortality, underscoring the crucial role of early detection [1–3]. Switzerland has neither a nationwide MSP nor MSPs in all federal states (cantons), but recent research also validated these beneficial findings in the Swiss context when comparing cantons with and without an MSP [4]. While the overall goal of MSPs is to detect BC as early as possible and thus to reduce mortality, BC may go undetected. For instance, this could be due to the limited ability to detect abnormalities in dense breast tissue, specific tumor development pathways that only manifest slowly and with minor signs, and radiologist errors in either visual perception or interpretation [5, 6]. Interval breast cancers (IBC) are cases where BC was diagnosed within the screening interval [7]. IBCs are concerning, as they are associated with more aggressive tumor characteristics, higher mortality rates, and require more frequently invasive treatments than screen-detected BC [7–9]. While the occurrence of IBCs is not completely avoidable due to the natural progression of cancers between screenings, minimizing their incidence by higher screening sensitivity is vital for improving the effectiveness of MSPs. Thus, the rate of IBC cases is a key quality indicator of MSPs [7, 10].

In general, the number of IBCs could be reduced by shortening the intervals (for women at higher risk) or by using other imaging modalities (e.g., MRI), which comes with workload increase, higher costs for an MSP and higher burden for affected women [11]. The advent of artificial intelligence (AI) presents a promising opportunity to enhance early detection of BC within MSPs without necessarily increasing workload. Multiple studies outlined the potential benefit of AI systems to improve the accuracy and/or efficiency of MSPs in BC detection [12–17]. However, few studies to date have tested the ability of AI to reduce IBCs [6, 14, 18]. Specifically, “the UK National Screening Committee review cited the lack of consistent evidence on the performance of AI algorithms in the detection of interval cancers as a major reason for not recommending the integration of AI into routine mammography screening workflows” [19, 20]. Further, neither the specific AI system has been tested for its potential to reduce IBCs, nor has such a study been conducted in Switzerland. Thus, our study aims to answer the research question: Can an AI system identify BC signs in IBC screening mammograms? Additionally, we presented the IBC screening mammograms independently to three radiologists to retrospectively evaluate whether there were any visible BC signs.

## Materials and methods

### Ethics approval

This retrospective cohort study was submitted to the Ethics Commission of Eastern Switzerland (EKOS–23/061). It was ruled to be a quality control study covered by the informed patient consent of the MSP. All data were pseudonymized by the Cancer Registry of Eastern Switzerland prior to analyses.

The study followed the STARD guideline for reporting diagnostic accuracy studies [21].

### Study population

The study included all IBC cases of women who participated in the MSP “donna” (double reading) in the Swiss cantons of St.Gallen and Grisons (10% of Swiss inhabitants) between 2010 and 2019. All women between 50 and 69 years of age in the cantons of St.Gallen (since 2010) and Grisons (since 2011) were invited every 2 years to voluntarily participate in the MSP, which is covered by the compulsory health insurance except for a co-payment of less than 20 USD [22]. A recorded tumor was classified as IBC when an invasive or in situ BC (ICD-10-codes: C50/D05 in the cancer registry) was diagnosed within 24 months after a normal (i.e., no breast cancer diagnosis, including cases that have been discussed in a consensus conference/further investigated) screening mammogram (similar as in Niraula et al [7]/the European guidelines [10]). Cancer data were retrieved from the databases of the Cancer Registries of Eastern Switzerland and Grisons-Glarus, documenting all cancer cases within their cantons.

### AI system

We utilized the AI system ProFound AI® from iCAD (version 3.1 for 2D FFDM), specifically the case score and risk score/category. The case score reflects the degree of confidence that a mammogram currently includes a BC compared to the training database on a scale from 0 (BC very unlikely) to 100 (BC very likely) [23]. The numerical risk score reflects the risk (probability) of being diagnosed with BC within the next 2 years. This forecasted risk is based on age, regional incidence data and numerous mammographic characteristics and is also expressed on a scale of 0 to 100 [24]. Additionally, the numerical risk score is used to classify a mammogram into one of four risk categories (as defined by vendor): low (< 0.15%), general (0.15–0.59%), moderate (0.6–1.59%), or high (≥ 1.6%) in reference to the average risk of a woman’s age.

### Sample generation and data processing

**Fig. 1:**
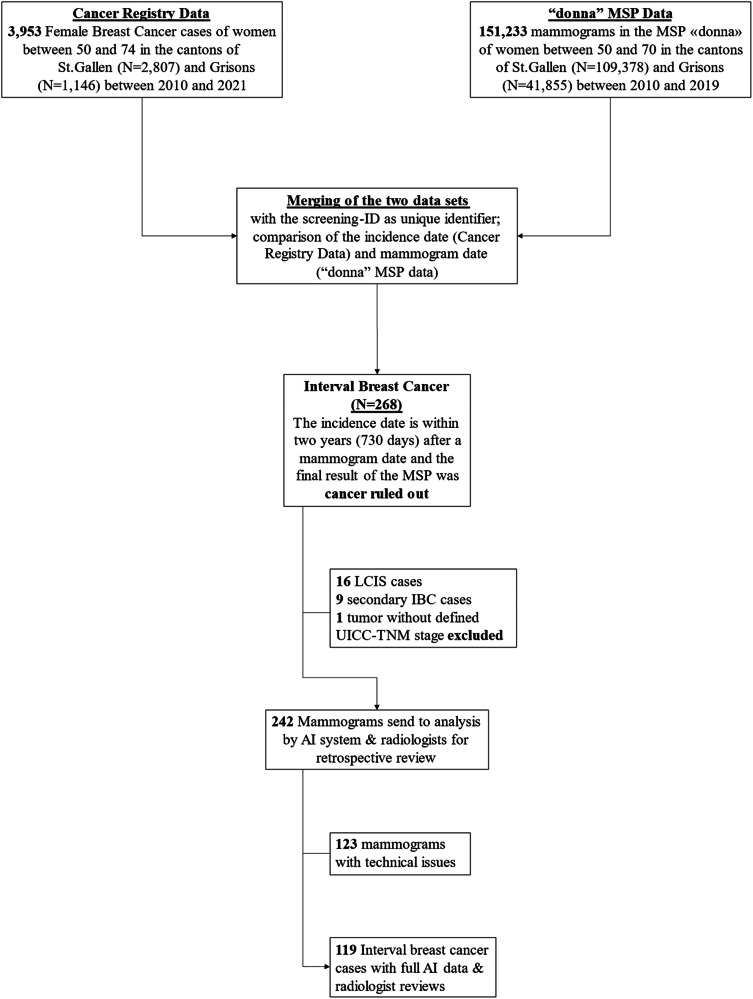
Derivation of the final data set. Some of the older mammography screening devices (until 2014) are not compatible with the AI system

From 151,233 women who participated in screening in the MSP “donna” from 2010 to 2019, 268 IBCs have been identified. Of the 268 IBCs, 242 IBC screening mammograms were independently provided to the radiologists and the AI system (after excluding LCIS cases, secondary IBC cases, and a tumor without a defined UICC-TNM stage). 119 IBC screening mammograms have been interpretable by the AI system (i.e., the other mammograms could not be analyzed because the stored mammograms have been performed by mammography screening devices that are not compatible with the AI system). Figure 1 shows the derivation of the final sample. This includes variables from the MSP, the cancer registry, and the outcomes from the AI system. MSP data included, for example, the year of screening, breast density (Breast Imaging Reporting and Data System [25] [BI-RADS] a to d), both radiologists’ reviews (R1 to R5), consensus conference conclusions (BC ruled out or further investigation), further conducted examinations (e.g., MRI), overall MSP outcome (BC detection/cancer ruled out) and self-declared information as family BC history (mother, sister or daughter diagnosed with BC). Further, cancer registry data included diagnosis and staging (e.g., ICD-10, TNM classifications), individual information (e.g., age at diagnosis), cancer biology information (e.g., Ki-67 proliferation index), treatment information (e.g., mastectomy), medical follow-up (e.g., recurrence), and vital status. Women with LCIS (i.e., D05.0) were excluded from the analysis as LCIS is a benign condition and not treated as a carcinoma [26].

### Retrospective evaluation of the IBC mammograms by radiologists (no AI involvement)

Three radiologists (two Swiss, one German, all with 10+ years’ experience, actively reviewing breast cancer screening mammograms in MSPs, and no exposure to AI in regular screening) were each provided with all IBC screening mammograms and 90 normal screening mammograms without BC that have been randomly selected by the cancer registry data analyst and mixed between the IBC mammograms to reduce the bias of radiologists, not to assume that they can always detect something. The blind review methodology, first looking at the screening mammogram without knowing the localization of the BC, is aligned with the European guidelines [10]. The radiologists independently assessed the mammograms, and whenever one or more radiologists assigned a mammogram with R3 or higher, the case was discussed during a consensus conference. Here, the screening mammograms were jointly reviewed and discussed, and a final decision was made on whether further examination was warranted due to visible abnormalities indicating malignancy. To classify the IBCs into the standardized categories (true interval, occult, minimal signs, false negative), the European guidelines require that the retrospective review evaluate first the screening mammogram and then the diagnostic mammogram. As we did not have access to the diagnostic mammograms, these categories could not be utilized. A reduced classification was sufficient to address our research question, distinguishing between “IBCs without retrospective abnormalities” and “potentially missed IBCs” if the consensus conference recommended further investigations.

### Statistical analyses

A descriptive table outlines patient, MSP, AI system output and cancer characteristics, stratified according to the “IBCs without retrospective abnormalities” and “potentially missed IBC” subgroups. ANOVA and Student’s *t*-test, as appropriate, highlight the significance of differences in patient-level variables, including tumor characteristics, between the subgroups. Diagrams visualize the distribution of case scores and risk classifications of the two subgroups, additionally stratified by breast density. A scatterplot chart illustrates the two key AI outputs in a graph, with the additional information on whether these IBC cases have been discussed in a consensus conference. Line graphs and respective sensitivity tables stress the share of IBC cases that would have been classified as abnormal based on the respective AI case score threshold/within the respective risk category. Results were considered significant at the 95% confidence level. All statistical analyses were performed in Stata 18.5 [27].

## Results

Table 1 shows that the women with IBC were on average 57.3 (standard deviation (SD): 5.4) years old, born in Switzerland (73.1%; *n* = 87), from the canton St.Gallen (80.7%; *n* = 96), and had a high breast density (BI-RADS c/d: 63:0%; *n* = 75). The retrospective review by radiologists of the screening mammograms resulted in 68.9% (*n* = 82) IBCs without retrospective abnormalities and 31.1% (*n* = 37) potentially missed IBCs. There were no significant differences in evaluated patient characteristics between the two subgroups. Potentially missed IBC received significantly higher radiologist reviews compared to the IBCs without retrospective abnormalities during the previous MSP (BI-RADS 3-5: 62.2% vs. 8.5%) and within the retrospective evaluations (BI-RADS 3-5: 100% vs. 51.2%; *p* < 0.05). During the previous screening, 40.5% (*n* = 15) of potentially missed IBCs (vs. 1.2% of IBCs without retrospective abnormalities) were investigated further (including some biopsies). The retrospective evaluation suggested further investigations for all potentially missed IBC cases.

**Table 1:** Patient and tumor characteristics

	All IBCs		IBCs without retrospective abnormalities		Potentially missed IBCs		Test for differences
	*N*	Mean (SD)/proportion	*N*	Mean (SD)/proportion	*N*	Mean (SD)/proportion	*p*-value
**Patient characteristics**							
Age	119	57.3 (5.4)	82	56.8 (5.0)	37	58.4 (6.2)	*p* = 0.15^g^
Birthplace (%)							*p* = 0.98^h^
Outside Switzerland	32	26.89%	22	26.83%	10	27.03%	
In Switzerland	87	73.11%	60	73.17%	27	72.97%	
Canton (%)							*p* = 0.67^h^
Grisons	23	19.33%	15	18.29%	8	21.62%	
St.Gallen	96	80.67%	67	81.71%	29	78.38%	
Breast density^a^							*p* = 0.20^h^
BI-RADS a	5	4.20%	2	2.44%	3	8.11%	
BI-RADS b	39	32.77%	27	32.93%	12	32.43%	
BI-RADS c	71	59.66%	49	59.76%	22	59.46%	
BI-RADS d	4	3.36%	4	4.88%	0	0.00%	
Family history of BC^b^							*p* = 0.19^h^
Positive	32	26.89%	25	30.49%	7	18.92%	
Negative	87	73.11%	57	69.51%	30	81.08%	
**Initial MSP results**							
Highest radiologist review^a^	119	32.8 (2.8)	82	23.11 (2.6)	37	54.13 (5.2)	*p* < 0.05^h^
R1	57	47.90%	51	62.20%	6	16.22%	
R2	32	26.89%	24	29.27%	8	21.62%	
R3	21	17.65%	5	6.10%	16	43.24%	
R4	8	6.72%	2	2.44%	6	16.22%	
R5	1	0.84%	0	0.00%	1	2.70%	
Consensus conferences							*p* < 0.05^h^
Yes	31	26.05%	8	9.76%	23	62.16%	
No	88	73.95%	74	90.24%	14	37.84%	
Further investigations^c^							*p* < 0.05^h^
Yes	16	13.45%	1	1.22%	15	40.54%	
No	103	86.55%	81	98.78%	22	59.46%	
**Retrospective radiologist evaluations**							
Highest radiologist review^d^							*p* < 0.05^h^
R1	0	0.00%	0	0.00%	0	0.00%	
R2	40	33.61%	40	48.78%	0	0.00%	
R3	18	15.13%	17	20.73%	1	2.70%	
R4	54	45.38%	24	29.27%	30	81.08%	
R5	7	5.88%	1	1.22%	6	16.22%	
Consensus conferences							*p* < 0.05^h^
Yes	64	53.78%	27	32.93%	37	100%	
No	55	46.22%	55	67.07%	0	0.00%	
Further investigations^e^							*p* < 0.05^h^
Yes	38	31.93%	1	1.22%	37	100%	
No	81	68.07%	81	98.78%	0	0.00%	
**AI output**							
Case scores	119	32.8 (30.0)	82	23.1 (23.8)	37	54.1 (31.6)	*p* < 0.05^g^
≥ 0 and < 10	39	32.77%	37	45.12%	2	5.41%	
≥ 10 and < 20	17	14.29%	12	14.63%	5	13.51%	
≥ 20 and < 30	12	10.08%	8	9.76%	4	10.81%	
≥ 30 and < 40	12	10.08%	9	10.98%	3	8.11%	
≥ 40 and < 50	9	7.56%	5	6.10%	4	10.81%	
≥ 50 and < 60	3	2.52%	2	2.44%	1	2.70%	
≥ 60 and < 70	8	6.72%	3	3.66%	5	13.51%	
≥ 70 and < 80	5	4.20%	2	2.44%	3	8.11%	
≥ 80 and < 90	5	4.20%	3	3.66%	2	5.41%	
≥ 90 and ≤ 100	9	7.56%	1	1.22%	8	21.62%	
Risk classification							*p* < 0.05^h^
Low	0	0.00%	0	0.00%	0	0.00%	
General	46	38.66%	39	47.56%	7	18.92%	
Moderate	41	34.45%	31	37.80%	10	27.03%	
High	30	25.21%	12	14.63%	18	48.65%	
n.a.	2	1.68%	0	0.00%	2	5.41%	
**Cancer data**							
Stage distribution (%)							*p* = 0.25^h^
In situ	8	6.72%	7	8.54%	1	2.70%	
I	41	34.45%	28	34.15%	13	35.14%	
II	43	36.13%	29	35.37%	14	37.84%	
III	15	12.61%	13	15.85%	2	5.41%	
IV	11	9.24%	4	4.88%	7	18.92%	
n.a.	1	0.84%	1	1.22%	0	0.00%	
Occurrence							*p* = 0.86^h^
Within 6 months	14	11.76%	10	12.20%	4	10.81%	
Between 7 and 12 months	30	25.21%	19	23.17%	11	29.73%	
Between 13 and 18 months	36	30.25%	28	34.15%	8	21.62%	
Between 19 and 24 months	39	32.77%	25	30.49%	14	37.84%	
Mean tumor size (mm)	119	20.9 (16.4)	82	22.0 (17.3)	37	18.5 (14.1)	*p* = 0.28^g^
Tumor size < 2 cm	62	52.10%	40	48.78%	22	59.46%	
Tumor size < 5 cm	110	92.44%	75	91.46%	35	94.59%	
Lymph node involvement							*p* = 0.06^h^
Positive (N1+)	27	22.69%	21	25.61%	6	16.22%	
Negative (N0)	68	57.14%	50	60.98%	18	48.65%	
n.a.	24	20.17%	11	13.41%	13	35.14%	
Grading^f^							*p* = 0.25^h^
Grade I	16	13.45%	9	10.98%	7	18.92%	
Grade II	55	46.22%	39	47.56%	16	43.24%	
Grade III	39	32.77%	26	31.71%	13	35.14%	
n.a.	9	7.56%	8	9.76%	1	2.70%	
Hormone receptor							*p* = 0.74^h^
Positive	96	80.67%	65	79.27%	31	83.78%	
Negative	20	16.81%	14	17.07%	6	16.22%	
n.a.	3	2.52%	3	3.66%	0	0.00%	
Estrogen receptors							*p* = 0.38^h^
Positive (≥ 1)	96	80.67%	65	79.27%	31	83.78%	
Negative	20	16.81%	14	17.07%	6	16.22%	
n.a.	3	2.52%	3	3.66%	0	0.00%	
Progesterone receptors							*p* = 0.39^h^
Positive (≥ 1)	80	67.23%	54	65.85%	26	70.27%	
Negative	35	29.41%	24	29.27%	11	29.73%	
n.a.	4	3.36%	4	4.88%	0	0.00%	
HER2 overexpression							*p* = 0.91^h^
Positive	24	20.17%	18	21.95%	6	16.22%	
Negative	89	74.79%	59	71.95%	30	81.08%	
n.a.	6	5.04%	5	6.10%	1	2.70%	
Triple negative	12	10.08%	9	10.98%	3	8.11%	*p* = 0.59^h^
Ki-67 proliferation index							*p* = 0.83^h^
Low (< 10%)	16	13.45%	9	10.98%	7	18.92%	
Medium (≥ 10%, < 25%)	44	36.97%	31	37.80%	13	35.14%	
High (≥ 25%)	50	42.02%	35	42.68%	15	40.54%	
n.a.	9	7.56%	7	8.54%	2	5.41%	

^a^ According to BI-RADS, the highest review from two initial readers, and some random cases are also evaluated by a third reader for training purposes

^b^ Mother, sister or daughter diagnosed with BC

^c^ Diverse further investigation options as another mammogram, ultrasound, MRI to biopsy

^d^ According to BI-RADS, the highest review from all three radiologists participating in the retrospective evaluation of IBC mammograms

^e^ Further investigations suggested by a retrospective consensus conference due to abnormal findings in screening mammogram

^f^ According to the Union for International Cancer Control

^g^ Student’s *t*-test

^h^ Anova

**Fig. 2:**
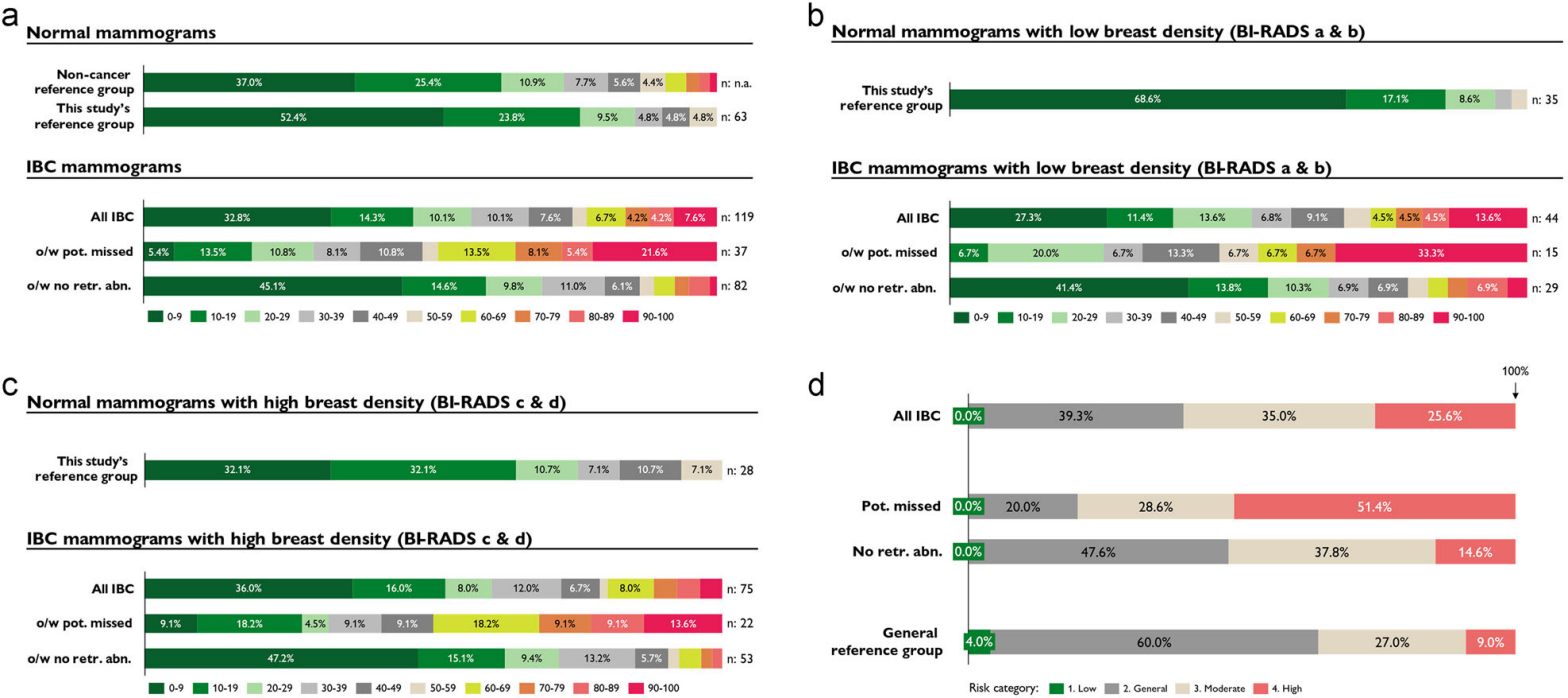
**a** Relative distribution of case scores. Source for non-cancer reference group: iCAD Inc. 2023 [24]. **b** Relative distribution of case scores: Low breast density subgroup. **c** Relative distribution of case scores: High breast density subgroup. **d** Relative distribution of risk categories. Source for general reference group: Eriksson et al [12]; General reference group including normal, screen-detected, and interval breast cancer screening mammograms from a representative Swedish screening population

The AI system rated the IBC screening mammograms with much higher case scores compared to a representative non-cancer reference group and the normal mammograms of this study (see Fig. 2a). The potentially missed IBCs received significantly higher case scores compared to the IBCs without retrospective abnormalities (mean case score: 54.1 (5.2) vs. 23.1 (2.6); *p* < 0.05; Table 1). Examining IBC mammograms by breast density of women, it is evident that case scores are higher for low breast density, especially for potentially missed IBCs with low breast density (see Fig. 2b, c). Further, the AI system assigned the IBC screening mammograms to higher risk categories compared to a general reference group (see Fig. 2d). The potentially missed IBCs have been assigned to significantly higher risk categories compared to the IBCs without retrospective abnormalities (high-risk category: 48.7% vs. 14.6%; *p* < 0.05; Table 1).

**Fig. 3:**
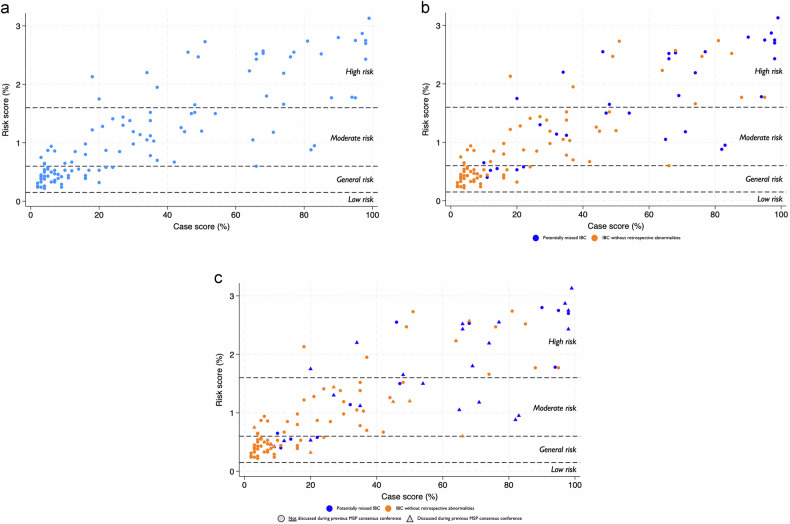
**a** Distribution of case and risk score for all IBC cases. **b** Distribution of case and risk score stratified by subgroup. **c** Distribution of case and risk score stratified by subgroup and consensus conference

The combined distribution of case and risk score (see Fig. 3a) highlights that many IBC cases are in the low to medium case and risk score area and shows a noticeable association between case and risk score, with some exceptions. 46.2% of all IBCs received a case score above 25, 25.2% above 50, and 13.4% above 75 (Table 2). Figure 3b outlines the distribution of case and risk score for the two subgroups, where it is evident that many IBCs without retrospective abnormalities have very low case and risk scores, whereas the potentially missed IBCs are spread widely from low to high case and risk scores. 73.0% of potentially missed IBCs (vs. 34.1% of IBCs without retrospective abnormalities) received a case score above 25, 51.4% (vs. 13.4%) above 50, and 29.7% (vs. 6.1%) above 75. In addition, Fig. 3c highlights visually by subgroup whether the individual IBC cases have been discussed in a consensus conference within the previous MSP. Here, it is visible that there is a considerable number of IBCs without retrospective abnormalities, as well as potentially missed IBCs that have a high case-/risk score that have not been discussed in a consensus conference before. Specifically, 54.8% of all IBC (60.9% potentially missed IBCs vs. 37.5% IBCs without retrospective abnormalities) received a case score above 50 and have been discussed during a consensus conference during the MSP; similarly, 22.6% of all IBC (39.1% vs. 0%) received a case score above 75 and have been discussed (Table 2).

**Table 2:** Accumulated IBC cases per case score threshold

	General distribution			o/w consensus conference			o/w further investigated		
Case scores	All IBCs (*n* = 119)	No retr. abn. (*n* = 82)	Pot. missed (*n* = 37)	All IBCs	No retr. abn.	Pot. missed	All IBCs	No retr. abn.	Pot. missed
**≥ 95**	5.9%	1.2%	16.2%	12.9%	0.0%	17.4%	18.8%	0.0%	20.0%
**≥ 90**	7.6%	1.2%	21.6%	12.9%	0.0%	17.4%	18.8%	0.0%	20.0%
**≥ 85**	9.2%	3.7%	21.6%	12.9%	0.0%	17.4%	18.8%	0.0%	20.0%
**≥ 80**	11.8%	4.9%	27.0%	19.4%	0.0%	26.1%	25.0%	0.0%	26.7%
**≥ 75**	13.4%	6.1%	29.7%	22.6%	0.0%	30.4%	31.3%	0.0%	33.3%
**≥ 70**	16.0%	7.3%	35.1%	29.0%	0.0%	39.1%	37.5%	0.0%	40.0%
**≥ 65**	21.8%	9.8%	48.6%	48.4%	25.0%	56.5%	50.0%	0.0%	53.3%
**≥ 60**	22.7%	11.0%	48.6%	48.4%	25.0%	56.5%	50.0%	0.0%	53.3%
**≥ 55**	22.7%	11.0%	48.6%	48.4%	25.0%	56.5%	50.0%	0.0%	53.3%
**≥ 50**	25.2%	13.4%	51.4%	54.8%	37.5%	60.9%	62.5%	100%	60.0%
**≥ 45**	31.1%	17.1%	62.2%	61.3%	50.0%	65.2%	68.8%	100%	66.7%
**≥ 40**	32.8%	19.5%	62.2%	61.3%	50.0%	65.2%	68.8%	100%	66.7%
**≥ 35**	38.7%	26.8%	64.9%	64.5%	50.0%	69.6%	68.8%	100%	66.7%
**≥ 30**	42.9%	30.5%	70.3%	67.7%	50.0%	73.9%	75.0%	100%	73.3%
**≥ 25**	46.2%	34.1%	73.0%	74.2%	62.5%	78.3%	75.0%	100%	73.3%
**≥ 20**	52.9%	40.2%	81.1%	83.9%	75.0%	87.0%	87.5%	100%	86.7%
**≥ 15**	60.5%	50.0%	83.8%	83.9%	75.0%	87.0%	87.5%	100%	86.7%
**≥ 10**	67.2%	54.9%	94.6%	87.1%	75.0%	91.3%	93.8%	100%	93.3%
**≥ 5**	84.9%	78.0%	100%	96.8%	87.5%	100%	100%	100%	100%

“No retr. abn.”: IBCs without retrospective abnormalities; “Pot. missed”: potentially missed IBCs; “o/w consensus conference”: IBCs that have been discussed within the previous MSP consensus conference; “o/w further investigated”: IBCs that have been further investigated within the previous MSP

## Discussion

Our study aims to assess whether an AI system can identify BC signs in IBC screening mammograms and thus reduce the number of IBCs in an MSP. Therefore, we retrospectively analyzed the IBC cases from the Swiss MSP “donna” from 2010 to 2019 using a retrospective radiologist review and an AI system. When reviewing the IBC characteristics of the study population, we found that they are in line with previous studies (e.g., high breast density) [5, 6, 28]. It is noteworthy that the radiologists observed abnormalities in 26.1% of all IBCs already during the previous screening, discussed these cases during a consensus conference, and ultimately investigated 13.5% of all IBCs further without detecting BC. In comparison, in the screening routine of the MSP “donna,” usually only around 10% of all cases are discussed in a consensus conference. Similarly, other international studies also found higher rates of consensus meetings for IBCs [29–31]. The radiologists’ retrospective assessments resulted in 68.9% IBCs without retrospective abnormalities and 31.1% potentially missed IBCs. Other studies come to similar shares of around 30% missed IBCs, but the specific proportion of IBCs retrospectively classified as missed significantly depends on the review methodology (e.g., blinded vs. fully informed review) [32, 33].

The distribution of case scores for all IBC cases indicates that the AI system identified abnormalities in a relevant portion of the IBC screening mammograms. In detail, the AI system assigned overall high case scores to potentially missed IBCs (51.4% with a case score above 50), where radiologists retrospectively also observed abnormalities. The high case scores for potentially missed IBCs highlight a high congruence where both the radiologists and the AI system detected abnormalities in the screening mammograms, even though these cases have not been identified in the MSP. In addition, the AI system recognized a fair share of IBCs without retrospective abnormalities (13.4% with a case score above 50), where no abnormalities were seen by the radiologists, indicating its complementary value. In practice, the AI system was recently introduced as a third reader in the MSP “donna,” with a case score threshold of 55, above which all cases are being discussed in a consensus conference (based on local historical data to discuss a maximum of 15% of all MSP cases in a consensus conference). When applying the case score threshold of 55 to our IBC population, 22.7% of the IBC cases get flagged by the AI system, and 51.6% of these cases were not discussed in a consensus conference during the previous screening. Thus, 11.7% of the IBC cases would now additionally be discussed in a consensus conference.

**Fig. 4:**
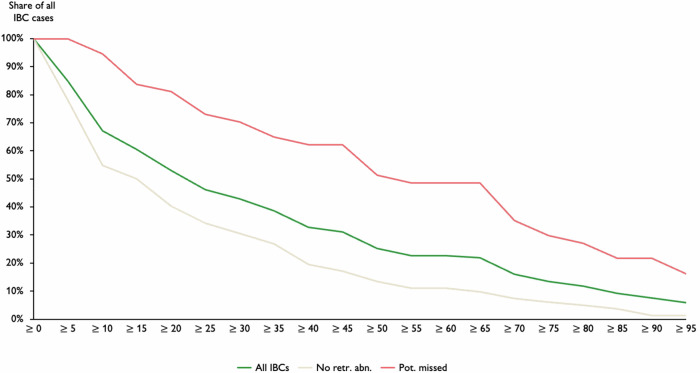
Share of IBC cases above a certain case score threshold

While there is only limited evidence so far, one retrospective study from Sweden concluded that their AI system (Transpara) might aid radiologists in detecting up to 19.3% of the IBCs at screening where the mammogram showed at least minimal signs of malignancy [6]. One study from Denmark tested different deployment scenarios of an AI system (Lunit) within an MSP and concluded that a triage-based approach provides the best performance metrics (e.g., accuracy, workload) when evaluating screen-detected and IBC screening mammograms [34]. Depending on the deployment scenario, between 13.3% (triage) and 19.5% (replacing one radiologist) of IBC cases would have been flagged by the respective AI system. Therefore, 25.2% of our IBCs with a case score above 50 are in line with these findings and at the upper end, primarily driven by potentially missed IBCs with overall much higher case scores (see Fig. 4).

The evaluation of the risk scores/-categories led to similar findings (see Table 3). Relative to a representative Swedish screening population, women with IBCs were categorized at much higher risk categories by the AI system (25.6% vs. 9% with high-risk) [12]. When comparing potentially missed IBCs with IBCs without retrospective abnormalities, the AI assigned significantly higher risk categories (51.4% vs. 14.6% within the high-risk category) to the potentially missed IBCs, aligning with the radiologists’ retrospective findings of abnormalities in these mammograms. One previous study identified that the same AI system as in our study and its risk forecasting component can predict around 30% of stage 2+ BCs in 6% of high-risk women, where no BC was detected during the MSP [12]. With 25.6% of all our IBCs being classified as high-risk, where later IBC was diagnosed across all stages, this figure is directionally in line with these findings. Another study retrospectively reviewed the screening mammograms by radiologists and a different AI system (Transpara) and found that there were 33% of IBCs that were classified as “true negative” and had been assigned the highest AI risk score [6]. Additionally, a study employed an AI system to analyze normal/negative MSP mammograms for BC risk. The ones with the highest risk underwent an additional MRI examination, resulting in a much higher efficiency in cancer detection compared to conventional density measurements and risk models [35].

**Table 3:** IBC cases per risk category

	General distribution			o/w consensus conference			o/w further investigated		
Risk category	All IBCs (*n* = 117)	No retr. abn. (*n* = 82)	Pot. missed (*n* = 35)	All IBCs (*n* = 31)	No retr. abn. (*n* = 8)	Pot. missed (*n* = 23)	All IBCs (*n* = 16)	No retr. abn. (*n* = 1)	Pot. missed (*n* = 15)
High	25.6%	14.6%	51.4%	41.9%	12.5%	52.2%	62.5%	0.0%	66.7%
Moderate	35.0%	37.8%	28.6%	35.5%	50.0%	30.4%	18.8%	100%	13.3%
General	39.3%	47.6%	20.0%	22.6%	37.5%	17.4%	18.8%	0.0%	20.0%

“No retr. abn.”: IBCs without retrospective abnormalities; “Pot. missed”: potentially missed IBCs; “o/w consensus conference”: IBCs that have been discussed within the previous MSP consensus conference; “o/w further investigated”: IBCs that have been further investigated within the previous MSP

The joint examination of both AI information and the radiologist assessments outlines that there are many IBCs where neither the AI system nor the radiologists could find evidence for BC in the screening mammograms. However, it also highlights that there are many IBC screening mammograms where both AI systems’ outputs (moderate to high case and risk score) flagged abnormalities/indicated risk. Moreover, there are some IBCs with just a moderate to high case score (but low to moderate risk category) or just a moderate to high-risk category (but low to moderate risk case score). Thus, it is valuable to consider both AI system information.

### Limitations

Our retrospective study design inherently limits our ability to draw causal conclusions and emphasizes the need for prospective studies. It is especially important to evaluate how the radiologists utilize the AI information in a consensus conference, where they do not see any abnormalities in the mammogram, and a decision needs to be made if/which further investigations (e.g., MRI, ultrasound, or contrast-enhanced mammography) should be conducted. Further, without the diagnostic mammogram or the detailed position of the diagnosed BC, we could not evaluate whether the AI-marked lesion corresponds to the later detected BC.

Future research needs to include a large representative sample of mammograms (i.e., screening-detected BC, IBC, normal mammograms) to substantiate the sensitivity and specificity of the AI system. This is important to define a practically relevant AI case-/risk score threshold, as only a fully representative sample can ensure that these thresholds are consistent with the overall optimization challenge (i.e., identification of the most possible BC cases at screening while avoiding excessive caseloads for consensus conferences/ultimately decreasing screening efficiency).

## Conclusion

Our research highlights that an AI system can identify BC signs in relevant portions of IBC screening mammograms and thus potentially reduce the number of IBCs in an MSP that currently does not utilize an AI system. It can do so by identifying most IBC cases that are retrospectively visible for radiologists but have been missed during the previous screening (without AI support), thereby acting as a safety net for human error. Additionally, it can identify some IBCs that are not visible to humans (IBCs without retrospective abnormalities). The specific proportion of avoidable IBC cases depends on the selected case score and/or the respective risk classification threshold, by which a case is flagged. Overall, our study demonstrates the potential positive impact of a different AI system in a new population, Switzerland. Further research is required to prospectively assess how AI information is utilized to ultimately diagnose BC, especially when humans do not see any abnormalities.

## Abbreviations

AI: Artificial intelligence
BC: Breast cancer
BI-RADS: Breast Imaging-Reporting and Data System of the American College of Radiology
IBC: Interval breast cancer
MSP: Mammography screening program
SD: Standard deviation
